# Supplementary material for: Critical spin liquid versus valence-bond glass in a triangular-lattice organic antiferromagnet
Source: Nat Commun. 2019 Jun 12;10:2561. doi: 10.1038/s41467-019-10604-3 (PMC6561973; doi:10.1038/s41467-019-10604-3)
Supplement: Supplementary file 1 — Supplemental Material [file 41467_2019_10604_MOESM1_ESM.pdf]

SUPPLEMENTARY NOTE 1: SPIN HAMILTONIAN

In this supplementary note, we present ab-initio calculations of the spin Hamiltonian for κ-(ET)$_2$Cu$_2$(CN)$_3$ (κ-Cu). In general, the magnetic interactions can be divided according to the number of spin operators appearing in each term $\mathcal{H}_{(n)} \sim \mathcal{O}(\mathbf{S}^n)$:

$$\mathcal{H} = \mathcal{H}_{(1)} + \mathcal{H}_{(2)} + \mathcal{H}_{(3)} + \mathcal{H}_{(4)} + ... \tag{1}$$

with $\mathcal{H}_{(1)}$ including the Zeeman operators, $\mathcal{H}_{(2)}$ including bilinear spin interactions, etc. We first present calculations of the interactions in the crystallographic coordinate system ($\mathcal{H}$) up to fourth order, and then describe the effects of the local coordinate transformation $\mathcal{H} \to \tilde{\mathcal{H}}$ introduced in the main text, which removes the anisotropic spin interactions at lowest order. We have previously[1] estimated the nearest neighbour bilinear couplings using a combination of hopping integrals obtained from ORCA at the PBE0/def2-VDZ level and exact diagonalization of small clusters of molecules. Using this approach, we have extended the calculations to estimate also longer-range couplings and higher order ring-exchange terms.

The Zeeman operator can be generally written:

$$\mathcal{H}_{(1)} = -\sum_i \mathbf{H} \cdot \mathbb{G}_i \cdot \mathbf{S}_i \tag{2}$$

in terms of the external field $\mathbf{H}$, local g-tensor $\mathbb{G}_i$ and spin operator $\mathbf{S}_i$ at dimer site i. Within each $P2_1/c$ unit cell, there are two molecular dimers, which are related by 2_1 screw axes and c-glide planes, shown in Supplementary Figure 1(a). As mentioned in the main text, the g-tensors may differ for the two dimer sublattices, labelled A and B, by symmetry. For this reason, it is useful to divide the g-tensor into uniform $\mathbb{G}_\mathrm{u}$ and staggered $\mathbb{G}_\mathrm{s}$ components, with:

$$\mathbb{G}_i = \mathbb{G}_\mathrm{u} + \eta_i \mathbb{G}_\mathrm{s} \tag{3}$$

$$\eta_i = \begin{cases} +1 & i \in \text{sublattice A} \\ -1 & i \in \text{sublattice B} \end{cases} \tag{4}$$

In order to estimate $\mathbb{G}_\mathrm{u}$ and $\mathbb{G}_\mathrm{s}$ for κ-Cu, we performed density functional theory calculations on isolated dimers at the PBE0/IGLO-III level using the ORCA package[2,3]. The molecular geometry was taken from the 5 K crystal structure reported in Supplementary Ref. 4. The principle axes (p, q, r) of the local g-tensors for the A and B sublattices are illustrated in

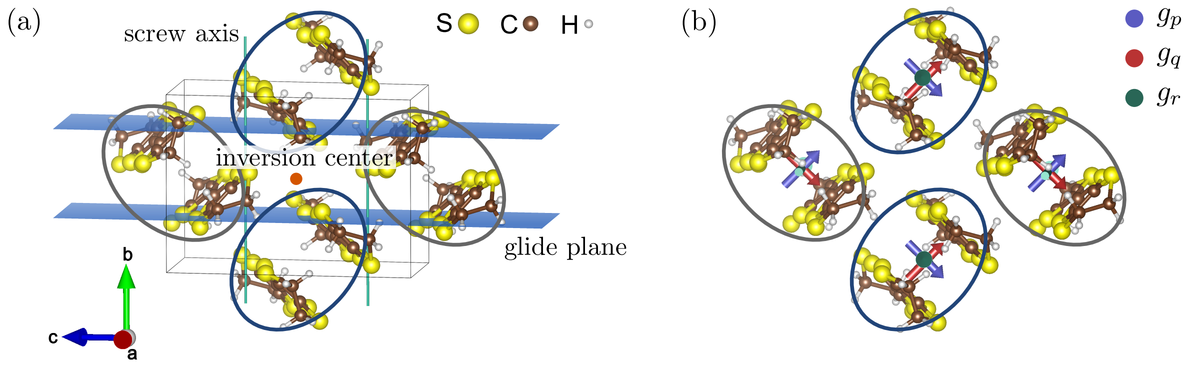

Supplementary Figure 1. **Symmetries in the $P2_1/c$ space group.** (a) Screw axes (green), glide planes (blue) and inversion center (orange) in the $P2_1/c$ space group, with two ET dimers per unit cell. (b) The principal axes (g_p, g_q, g_r) are shown in the two sublattices, labelled A and B.

Supplementary Figure 1(b). The largest value $g_r = 2.010$ corresponds to the long axis of the molecules, while the second largest $g_q = 2.008$ lies along the axis connecting the two molecules within each dimer. The final value of $g_p = 2.002$ was found for the third principal axis. In the crystallographic (a, b, c^*) coordinates, the uniform and staggered tensors are estimated as:

$$\mathbb{G}_{\mathrm{u}} = \begin{pmatrix} 2.010 & 0 & -8 \cdot 10^{-4} \\ 0 & 2.005 & 0 \\ 7 \cdot 10^{-4} & 0 & 2.005 \end{pmatrix}, \tag{5}$$

and:

$$\mathbb{G}_{\mathrm{s}} = \begin{pmatrix} 0 & -4 \cdot 10^{-4} & 0 \\ -6 \cdot 10^{-4} & 0 & -26 \cdot 10^{-4} \\ 0 & -25 \cdot 10^{-4} & 0 \end{pmatrix}. \tag{6}$$

At second order in the spin operators, the bilinear interactions can be generally written:

$$\mathcal{H}_{(2)} = \sum_{ij} J_{ij}\ \mathbf{S}_i \cdot \mathbf{S}_j + \mathbf{D}_{ij} \cdot (\mathbf{S}_i \times \mathbf{S}_j) + \mathbf{S}_i \cdot \Gamma_{ij} \cdot \mathbf{S}_j \tag{7}$$

where J_{ij} describes the Heisenberg coupling, $\mathbf{D}_{ij}$ is the Dzyaloshinskii-Moriya vector, and Γ_{ij} is a traceless symmetric tensor describing the pseudo-dipolar interaction. We label the unique interactions according to Supplementary Figure 2(a). For example, the anisotropic triangular lattice of nearest neighbour bonds is composed of J and J' interactions, while longer range second neighbour couplings are labelled J'' and J'''.

(g_p, g_q, g_r)	J	J'	J''	J'''	(D_a, D_b, D_{c^*})	K_h	K_v	K_d	K'_h	K'_v	K'_d	$J_{\chi,(1\mathrm{T})}$
$(2.002, 2.008, 2.010)$	228	268	9.5	5.1	$(3.30, 0.94, 0.99)$	16.5	13.6	-21.3	17.0	17.7	-20.5	-0.04

Supplementary Table 1. **Computed Hamiltonian Parameters.** g-tensor contribution along the principal axes (p, q, r) and computed magnetic exchange interactions in K with respect to (a, b, c^*), illustrated in Supplementary Figs. 1(b) and 2.

Within the $P2_1/c$ space group, the presence of a crystallographic inversion center forbids a DM interaction between sites i, j belonging to the same sublattice (i.e. $\mathbf{D}'_{ij} = 0$). Between different sublattices, $\mathbf{D}_{ij}$ is finite. By symmetry, the signs of the a and c^* axes components D_a and D_{c^*} are staggered with $k = (\pi, \pi)$ periodicity with respect to the square lattice bonds, as shown in Supplementary Figure 2(b). These components are responsible for the emergence of a small canted ferromagnetic moment in the magnetically ordered (π, π)-Néel phase of the related salt κ-$(ET)_2Cu(N(CN)_2)Cl$. In contrast, the b-axis component D_b has a striped periodicity. This component does not couple to any of the magnetic states expected to be relevant for κ-phase salts; we have therefore ignored this component in first approximation.

At this point, it is convenient to introduce a site-dependent coordinate transformation discussed in the main text, and by e.g. Shekhtman et al.[5] Employing this transformation, it is possible to completely "gauge" away some components of the anisotropic interactions satisfying particular symmetries. In particular, if the DM vectors sum to zero around all closed loops on the lattice, it is possible to make site-dependent transformations of the spin coordinates that simultaneously eliminate all leading order anisotropic contributions to $\{\mathcal{H}_{(n)}\}$ for $n > 1$.

The proof of this possibility is rooted in the microscopic form of the hopping Hamiltonian of the underlying electronic system. It is convenient to express the hopping Hamiltonian in terms of the spinor operators $\mathbf{c}^\dagger = (\, c_\uparrow^\dagger \; c_\downarrow^\dagger)$ as:

$$\mathcal{H}_{\text{hop}} = \sum_{ij} \mathrm{e}^{ia_{ij}} \mathbf{c}_i^\dagger \mathbb{T}_{ij} \mathbf{c}_j \tag{8}$$

$$\mathbb{T}_{ij} = \left(t_{ij} \mathbb{I}_{2\times 2} + \frac{i}{2} \boldsymbol{\lambda}_{ij} \cdot \boldsymbol{\sigma} \right) \tag{9}$$

Here, $\mathbb{I}_{2\times 2}$ is the 2×2 identity matrix, t_{ij} is the spin-diagonal hopping, while off-diagonal hopping $\boldsymbol{\lambda}_{ij}$ arises as a result of spin-orbit coupling. The above restriction on the sum

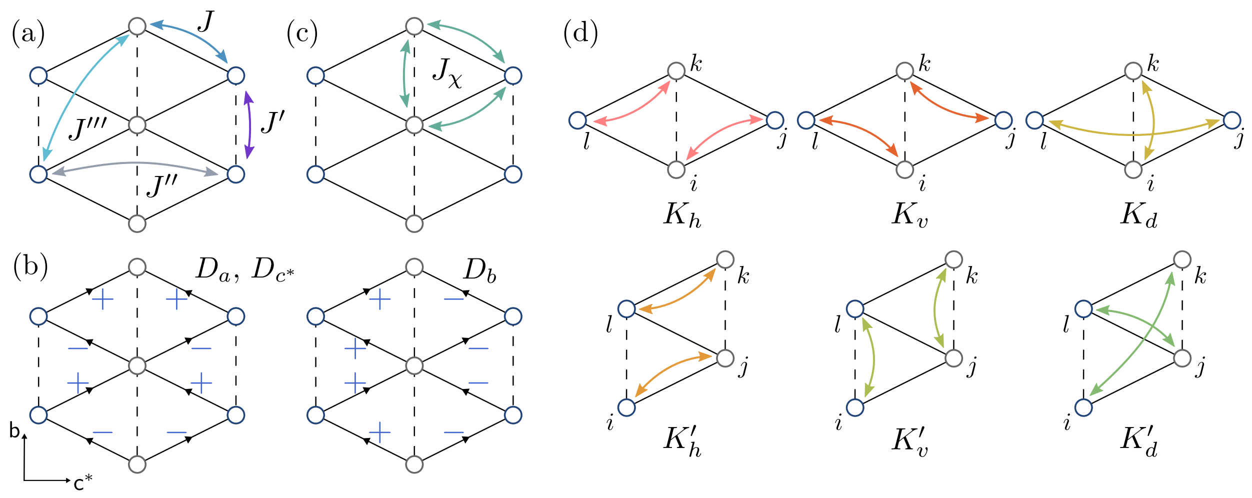

Supplementary Figure 2. **Definition of magnetic exchange parameters on anisotropic triangular lattice.** Each lattice point represents an ET dimer. (a) Heisenberg exchange parameters. (b) Pattern of a, c^* and b components of the DM interaction. (c) Three-spin scalar chiral exchange parameter on a three-site plaquette. (d) Definition of ring exchange parameters on the two distinct four site plaquettes in the anisotropic triangular lattice. As an example the spin Hamiltonian contains a term $K_v(\mathbf{S}_i \cdot \mathbf{S}_l)(\mathbf{S}_j \cdot \mathbf{S}_k)$ on a plaquette with one dashed and four solid bonds.

of DM-vectors around any closed loop is equivalent to restricting the product of hopping matrices around any closed path to be a multiple of the identity matrix:

$$\mathbb{T}_{ij}\mathbb{T}_{jk}...\mathbb{T}_{lm}\mathbb{T}_{mi} = C\ \mathbb{I}_{2\times 2} \tag{10}$$

We then consider making site-dependent spin rotations, which transform the operators as:

$$\tilde{\mathbf{c}}_i = \mathrm{e}^{i\mathbf{v}_i\cdot\boldsymbol{\sigma}}\mathbf{c}_i \tag{11}$$

in terms of some arbitrary vector $\mathbf{v}_i$. The transformed hopping matrices are then:

$$\tilde{\mathbb{T}}_{ij} = \mathrm{e}^{-i\mathbf{v}_i\cdot\boldsymbol{\sigma}}\mathbb{T}_{ij}\mathrm{e}^{i\mathbf{v}_j\cdot\boldsymbol{\sigma}} \tag{12}$$

The question of interest is whether we can define a transformation, defined by a specific set of $\{\mathbf{v}_i\}$, such that $\tilde{\mathbb{T}}_{ij} = \tilde{t}_{ij}\mathbb{I}_{2\times 2}$ on every bond. In fact, the restriction of Supplementary Eq. (10) guarantees this possibility. To see this, consider starting at site i, and making a string of site-dependent transformations at sites $j, k, ...$ to bring $\tilde{\mathbb{T}}_{ij}, \tilde{\mathbb{T}}_{jk}, ...$ into diagonal

form:

$$\tilde{\mathbb{T}}_{ij} = \mathbb{T}_{ij}\mathrm{e}^{i\mathbf{v}_j\cdot\boldsymbol{\sigma}} = \tilde{t}_{ij}\ \mathbb{I}_{2\times 2} \tag{13}$$

$$\tilde{\mathbb{T}}_{jk} = \mathrm{e}^{-i\mathbf{v}_j\cdot\boldsymbol{\sigma}}\mathbb{T}_{jk}\mathrm{e}^{i\mathbf{v}_k\cdot\boldsymbol{\sigma}} = \tilde{t}_{jk}\ \mathbb{I}_{2\times 2} \tag{14}$$

This process can be repeated indefinitely until the loop is about to be closed. The global transformation is consistent only if the string of transformations is compatible with the last $\tilde{\mathbb{T}}_{mi}$ also being proportional to the identity. Since the product is invariant:

$$\mathbb{T}_{ij}\mathbb{T}_{jk}...\mathbb{T}_{lm}\mathbb{T}_{mi} = \tilde{\mathbb{T}}_{ij}\tilde{\mathbb{T}}_{jk}...\tilde{\mathbb{T}}_{lm}\tilde{\mathbb{T}}_{mi}, \tag{15}$$

it holds that:

$$\tilde{\mathbb{T}}_{mi} = C\left(\prod_{i\to m}\frac{1}{\tilde{t}_{ij}}\right)\mathbb{I}_{2\times 2}. \tag{16}$$

Therefore, the final hopping matrix is automatically made diagonal by this string of transformations, provided Supplementary Eq. (10) holds. This completes the proof for the existence of a transformation that sets all $\tilde{\lambda}_{ij} \to 0$. The practical implication is that SOC effects can be completely gauged away already at the level of the underlying hopping Hamiltonian, from which the spin couplings are derived. As a result, all interactions appearing in the transformed spin Hamiltonians $\{\tilde{\mathcal{H}}_{(n)}\}$ for $n > 1$ must take an isotropic form.

In the case of a staggered (π, π) pattern, (as for the components D_a and D_{c^*} in κ-Cu), the local transformations $\mathbf{S} \to \tilde{\mathbf{S}}$ that eliminate the anisotropic couplings consist of rotations around $\mathbf{D}$ by the canting angle $\phi_i = 1/2\,\eta_i \arctan(|\mathbf{D}_{ij}|/J_{ij})$. Here, η_i is defined as in Supplementary Eq. (4). This rotation is illustrated in the left panel with $H = 0$ of Supplementary Figure 3. To leading orders, the pseudo-dipolar tensor can be expressed in terms of the DM vector as $\Gamma \propto \mathbf{D} \otimes \mathbf{D}$. In this limit, Γ is also exactly cancelled, leaving only the isotropic Heisenberg term:

$$\mathcal{H}_{(2),\mathrm{eff}} = \sum_{ij} \tilde{J}_{ij}\ \tilde{\mathbf{S}}_i \cdot \tilde{\mathbf{S}}_j \tag{17}$$

For small canting angles, i.e. weak spin-orbit coupling, we can work with the approximations $\cos\phi_i \approx 1$ and $\sin\phi_i \approx |\mathbf{D}_{ij}|/2J_{ij}$. This leads to $\tilde{J}_{ij} \approx J_{ij}$.

The utility of such a transformation is that it transfers the explicitly anisotropic terms to the Zeeman Hamiltonian, which contains then a uniform and staggered contribution:

$$\mathcal{H}_{\mathrm{Zee,eff}} = -\mu_{\mathrm{B}}\sum_i (\mathbf{H}_{\mathrm{u}} + \eta_i\mathbf{H}_{\mathrm{s}}) \cdot \tilde{\mathbf{S}}_i. \tag{18}$$

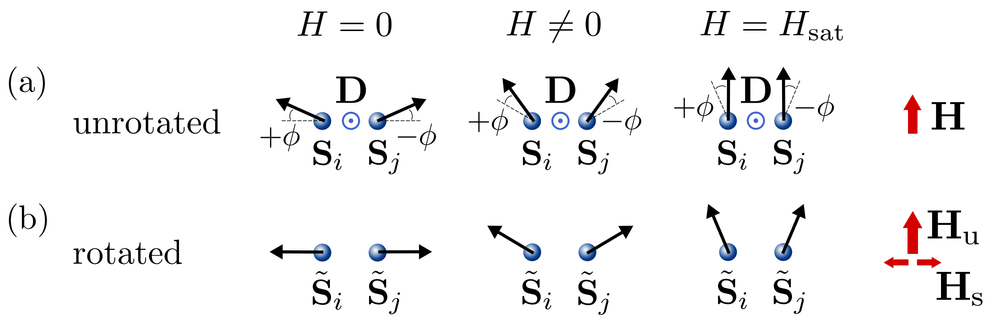

Supplementary Figure 3. **Gauging away anisotropic exchange terms.** Illustrated are the spins for zero, finite and saturation field in (a) the unrotated framework and (b) the rotated framework. The effective staggered field $\mathbf{H}_\mathrm{s}$ in the rotated framework is orthogonal to the uniform field $\mathbf{H}_\mathrm{u}$.

For small canting angles, the two field terms become:

$$\mathbf{H}_\mathrm{u} = \mathbb{G}_\mathrm{u}^\mathrm{T} \cdot \mathbf{H} \qquad \text{and} \qquad \mathbf{H}_\mathrm{s} = (\mathbb{G}_\mathrm{s} + \mathbb{R})^\mathrm{T} \cdot \mathbf{H}, \tag{19}$$

where we introduced the matrix:

$$\mathbb{R} = \frac{1}{2J} \mathbb{G}_\mathrm{u} \cdot \begin{pmatrix} 0 & D_{c^*} & 0 \\ -D_{c^*} & 0 & D_a \\ 0 & -D_a & 0 \end{pmatrix} \tag{20}$$

In the main text, these total terms are discussed as the total g-tensors in the rotated framework, which are for small canting angles:

$$\tilde{\mathbb{G}}_\mathrm{u} = \mathbb{G}_\mathrm{u} \qquad \text{and} \qquad \tilde{\mathbb{G}}_\mathrm{s} = \mathbb{G}_\mathrm{s} + \mathbb{R}. \tag{21}$$

Note that due to the structure of $\mathbb{R}$ and of the g-tensors Supplementary Eq. (5) and Supplementary Eq. (6) the effective uniform and staggered field are orthogonal. This is relevant for the scaling behaviour of the field-induced uniform and staggered magnetization, introduced in the main text.

Similar transformations can be applied to study the 3-spin interactions $\tilde{\mathcal{H}}_{(3)}$. Since any product of three spins at different sites is odd under time-reversal, such interactions are forbidden at zero field. However, as mentioned in the main text, a finite magnetic flux through the 3-site plaquettes can give rise to finite contributions to $\tilde{\mathcal{H}}_{(3)}$ that scales with odd powers of $|\mathbf{H}|$. These effects can be treated through the minimal coupling of the moving electron to the so-called Peierls phase $a_{ij} = \frac{q}{\hbar}\int_i^j \mathbf{A} \cdot \mathrm{d}\mathbf{l}$ in the transformed hopping Hamiltonian

$$\tilde{\mathbb{T}}_{ij} \to \mathbf{c}_i^\dagger \mathrm{e}^{i a_{ij}} \tilde{\mathbb{T}}_{ij} \tag{22}$$

This gives rise to odd order contributions in perturbation theory with a dependence on $\Phi = \oint_{\partial\mathcal{S}} \mathbf{A} \cdot \mathrm{d}\mathbf{l}$. This quantity is independent of the local spin coordinates, and therefore is invariant under the transformation described above.

The dominant three-spin term, illustrated in Supplementary Figure 2(c), is the so-called scalar spin chirality term,

$$\mathcal{H}_{(3),\text{eff}} = \frac{1}{S} \sum_{\langle ijk \rangle} \tilde{J}_{\chi}^{ijk} \; \tilde{\mathbf{S}}_i \cdot (\tilde{\mathbf{S}}_j \times \tilde{\mathbf{S}}_k). \tag{23}$$

with the exchange term given by (up to order t^3):

$$J_{\chi}^{(3)} = 24 \frac{t_{ij} t_{jk} t_{ki}}{U^2} \sin \Phi, \tag{24}$$

where Φ is proportional to the magnetic flux enclosed by the triangular plaquette $\langle ijk \rangle$. With the assumption of a homogeneous magnetic field ($\mathbf{A} = \frac{1}{2}\mathbf{r} \times \mathbf{H}$) we may use the approximation

$$\Phi = \frac{q}{\hbar} \mu_{\mathrm{B}} A_{\text{triangle}} (\mathbf{H} \cdot \mathbf{n}), \tag{25}$$

where $\mathbf{n}$ is the out-of-plane unit vector and A_{triangle} the area formed by the triangular plaquette. Numerical estimates for J_{χ}^{ijk} as a function of field, are shown in Supplementary Figure 4, and given in Supplementary Table 1 for $H = 1\,\mathrm{T}$. Following our previous approach, we performed exact diagonalization of the extended Hubbard Hamiltonian and projection on the corresponding low energy subspace on clusters of up to eight molecules. The hopping parameters used in the Hubbard picture were calculated with the ORCA package[2] and the two-particle parameters were chosen the same as in Supplementary Ref. 1 with a Hubbard repulsion $U = 0.55\,\mathrm{eV}$, a Hund's coupling $J_{\mathrm{H}} = 0.2\,\mathrm{eV}$, and a nearest neighbour Hubbard repulsion $V = 0.15\,\mathrm{eV}$. Here, we considered the largest term, corresponding to triangles with two J-bonds, and one J'-bond. In the limit of small fluxes $\sin \Phi \approx \Phi$, so that the exchange term depends for $\mathcal{O}(t^3)$ linearly on the field.

For convenience, it is useful to write the Hamiltonian in analogy with a Zeeman term:

$$\mathcal{H}_{(3),\text{eff}} = -\mu_{\mathrm{B}} \; (\mathbf{H} \cdot \mathbf{n}) \sum_{\langle ijk \rangle} j_{\Phi} \; \tilde{\mathbf{S}}_i \cdot (\tilde{\mathbf{S}}_j \times \tilde{\mathbf{S}}_k). \tag{26}$$

in terms of the unitless plaquette parameter:

$$j_{\Phi} = -\frac{1}{S} \frac{q}{\hbar} \frac{A_{\text{triangle}}}{\Phi} \; \tilde{J}_{\chi}^{ijk}, \tag{27}$$

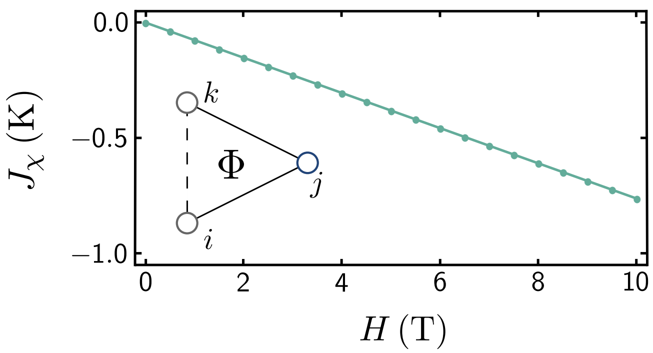

Supplementary Figure 4. **Exchange term of scalar spin chirality as a function of field.** The linear dependence on $H \propto \Phi$ is therefore confirmed numerically.

which is defined for each closed triangle plaquette $\langle ijk \rangle$. For the triangle pictured in Supplementary Figure 4, we estimated $j_\Phi \approx 0.039$. As noted in the main text, the operator $\tilde{\mathbf{S}}_i \cdot (\tilde{\mathbf{S}}_j \times \tilde{\mathbf{S}}_k)$ is isotropic, but these interactions provide explicit contributions to the magnetic torque through the appearance of $(\mathbf{H} \cdot \mathbf{n})$ in the coupling.

Finally, we have also considered higher order 4-spin ring-exchange couplings:

$$\mathcal{H}_{(4),\text{eff}} = \frac{1}{S^2} \sum_{\langle ijkl \rangle} \tilde{K}_{ijkl} (\tilde{\mathbf{S}}_i \cdot \tilde{\mathbf{S}}_j)(\tilde{\mathbf{S}}_k \cdot \tilde{\mathbf{S}}_l). \tag{28}$$

The distinct four-site plaquette are labelled according to a horizontal (K_h), vertical (K_v) and diagonal (K_d) interaction (shown in Supplementary Figure 2(d)). In previous works in which the ring-exchange terms have been considered, the approximation has been typically taken that $K_h = K_v = K_d$ and $K' = \frac{J'}{J} K$[6,7]. However, these relations are not enforced by symmetries. Interestingly, as shown in Supplementary Table 1, we find that such relations do not hold when considering the full electronic structure of the dimers. While the effects of such terms on the ground state are a matter of intense study, the isotropic ring-exchange terms do not explicitly contribute to the torque, and therefore may be the subject of future studies.

SUPPLEMENTARY NOTE 2: GENERALIZED TORQUE EXPRESSIONS

As mentioned in the main text, in general the magnetic torque is the derivative of the energy $E = \langle \mathcal{H} \rangle$ with respect to a reference angle θ:

$$\tau = \frac{\mathrm{d}\langle \mathcal{H} \rangle}{\mathrm{d}\theta} \tag{29}$$

This expression holds strictly in the $T \to 0$ limit, as entropic contributions are omitted for simplicity. After employing the site-dependent rotations described in Supplementary Note 1, the only terms in the Hamiltonian contributing to the magnetic torque are the uniform and staggered Zeeman terms and chiral 3-spin interactions. Here, we show the derivation of the bulk torque contribution for the uniform Zeeman term explicitly.

In terms of the uniform g-tensor $\tilde{\mathbb{G}}_{\mathrm{u}}$ and laboratory field $\mathbf{H}$, the uniform Zeeman Hamiltonian is:

$$\mathcal{H}_{\mathrm{Zee}} = -\mu_{\mathrm{B}} \mathbf{H} \cdot \tilde{\mathbb{G}}_{\mathrm{u}} \cdot \left(\sum_i \tilde{\mathbf{S}}_i \right). \tag{30}$$

In general, we assume that $\langle \sum_i \tilde{\mathbf{S}}_i \rangle = 0$ at zero field. In the presence of a finite field, there are several subtleties that arise due to anisotropy in $\tilde{\mathbb{G}}_{\mathrm{u}}$. For example, the Zeeman energy is minimized when the spins $\tilde{\mathbf{S}}_i$ are parallel to the effective field given by:

$$\mathbf{H}_{\mathrm{eff,u}} = \tilde{\mathbb{G}}_{\mathrm{u}}^{\mathrm{T}} \cdot \mathbf{H}, \tag{31}$$

where $\mathbb{G}^{\mathrm{T}}$ denotes the transpose of $\mathbb{G}$. As a result, we may write:

$$\left\langle \sum_i \tilde{\mathbf{S}}_i \right\rangle = \chi_u \ \mathbf{H}_{\mathrm{eff},u}, \tag{32}$$

in terms of a general susceptibility χ_{u}. Since the Hamiltonian governing the response of the spins $\tilde{\mathbf{S}}_i$ is otherwise isotropic, and we consider a regime with no spontaneously broken symmetry, the susceptibility χ_{u} is isotropic with respect to the effective field, and therefore depends only on the magnitude $|\mathbf{H}_{\mathrm{eff,u}}|$. We therefore assume that the susceptibility scales as a power law in terms of the magnitude of the effective field:

$$\chi_{\mathrm{u}} = \tilde{\chi}_{0,\mathrm{u}} \ |\mathbf{H}_{\mathrm{eff,u}}|^{-\zeta_{\mathrm{u}}} \tag{33}$$

Combining these expressions, the uniform Zeeman energy is given by:

$$\langle \mathcal{H}_{\text{Zee,eff,u}} \rangle = -\mu_{\text{B}}\, \mathbf{H} \cdot \tilde{\mathbb{G}}_{\text{u}} \cdot \left\langle \sum_i \tilde{\mathbf{S}}_i \right\rangle \tag{34}$$

$$= -\mu_{\text{B}}\, \tilde{\chi}_{0,\text{u}}\, |\mathbf{H}_{\text{eff,u}}|^{-\zeta_{\text{u}}} \left(\mathbf{H} \cdot \tilde{\mathbb{G}}_{\text{u}} \cdot \tilde{\mathbb{G}}_{\text{u}}^{\text{T}} \cdot \mathbf{H} \right) \tag{35}$$

$$= -\mu_{\text{B}}\, \tilde{\chi}_{0,\text{u}}\, |\mathbf{H}_{\text{eff,u}}|^{2-\zeta_{\text{u}}} \tag{36}$$

To compute the torque, we need to take the angular derivative of this expression. The θ-dependence arises from the norm of the effective field. For the derivative of the norm we use the following relation:

$$\frac{\text{d}}{\text{d}\theta} |\mathbf{H}_{\text{eff,u}}(\theta)|^{\alpha} = \alpha |\mathbf{H}_{\text{eff,u}}|^{\alpha-2}\, \frac{\text{d}\mathbf{H}_{\text{eff,u}}}{\text{d}\theta} \cdot \mathbf{H}_{\text{eff,u}}. \tag{37}$$

The torque we then obtain is given by:

$$\tau_{\text{u}}(\theta) = \frac{-\mu_{\text{B}}(2-\zeta_{\text{u}})\tilde{\chi}_{0,\text{u}} H^{2-\zeta_{\text{u}}}}{|\mathbb{G}_{\text{u}}^{\text{T}} \cdot \mathbf{h}|^{\zeta_{\text{u}}}} \left(\frac{\text{d}\mathbf{h}}{\text{d}\theta} \cdot \tilde{\mathbb{G}}_{\text{u}} \cdot \tilde{\mathbb{G}}_{\text{u}}^{\text{T}} \cdot \mathbf{h} \right). \tag{38}$$

where $\mathbf{h}$ is a unit vector in the direction of the laboratory field $\mathbf{H}$, and H is the magnitude of the laboratory field $H = |\mathbf{H}|$. In the main text, this expression is simplified by separating the H and θ dependencies:

$$\frac{\tau(\theta)}{H^2} = \tilde{\chi}_{\text{u}}(H) f_{\text{u}}(\theta) \tag{39}$$

$$\tilde{\chi}_{\text{u}}(H) = \mu_{\text{B}}(2-\zeta_{\text{u}})\tilde{\chi}_{0,\text{u}} H^{-\zeta_{\text{u}}} \tag{40}$$

$$f_{\text{u}}(\theta) = -\frac{1}{|\tilde{\mathbb{G}}_{\text{u}}^{\text{T}} \cdot \mathbf{h}|^{\zeta_{\text{u}}}} \left(\frac{\text{d}\mathbf{h}}{\text{d}\theta} \cdot \tilde{\mathbb{G}}_{\text{u}} \cdot \tilde{\mathbb{G}}_{\text{u}}^{\text{T}} \cdot \mathbf{h} \right) \tag{41}$$

Analogous expressions follow for the staggered and chiral contributions to the torque.

It is useful to see that these expressions reproduce the conventional $\sin 2\theta$ dependence in the case when $\zeta = 0$. To show this, we consider the torque in the $a - c^*$ plane, with θ being the angle between $\mathbf{H}$ and a within this plane. In this case, the corresponding torque is:

$$\tau_{a\text{-}c^*}(\theta) = -\tilde{\chi}_{\text{u}} \left[\frac{\text{d}}{\text{d}\theta} \begin{pmatrix} H\cos\theta \\ 0 \\ H\sin\theta \end{pmatrix} \right] \cdot \tilde{\mathbb{G}}_{\text{u}} \cdot \tilde{\mathbb{G}}_{\text{u}}^{\text{T}} \cdot \begin{pmatrix} H\cos\theta \\ 0 \\ H\sin\theta \end{pmatrix} \tag{42}$$

$$= \tilde{\chi}_{\text{u}} H^2 \begin{pmatrix} \sin\theta \\ 0 \\ -\cos\theta \end{pmatrix} \cdot \tilde{\mathbb{G}}_{\text{u}} \cdot \tilde{\mathbb{G}}_{\text{u}}^{\text{T}} \cdot \begin{pmatrix} \cos\theta \\ 0 \\ \sin\theta \end{pmatrix} \tag{43}$$

Assuming the g-tensor is diagonal in the $a - c^*$ coordinates, this gives:

$$\frac{\tau_{a\text{-}c^*}(\theta)}{H^2} = \mu_{\mathrm{B}}\ \tilde{\chi}_{0,\mathrm{u}}\ (g_{aa}^2 - g_{c^*c^*}^2)\sin(2\theta) \tag{44}$$

SUPPLEMENTARY NOTE 3: IMPURITY SCALING

In this supplementary note, we present the derivation of the approximate scaling expressions for the impurity contributions to the magnetic torque discussed in the main text. Generically, the coupling of such "orphan spins" to the external field $\mathbf{H}$ is governed by the Zeeman Hamiltonian:

$$\mathcal{H}_{\mathrm{Zee,I}} = -\mu_{\mathrm{B}} \sum_m \mathbf{H} \cdot \tilde{\mathbb{G}}_{\mathrm{I},m} \cdot \tilde{\mathbf{S}}_{\mathrm{I},m} \tag{45}$$

where $\tilde{\mathbb{G}}_{\mathrm{I},m}$ is the effective impurity g-tensor.

The response of the randomly coupled orphan spins can be understood with reference to the "strong disorder renormalization group" (SDRG) approach[8–13] to studying problems with quenched disorder. In the application to spin systems, the central quantity is the distribution of exchange couplings $\rho(J)$. An initial energy scale Ω is set by the strongest interaction within the network, which couples impurity spins $\tilde{\mathbf{S}}_{\mathrm{I},1}$, and $\tilde{\mathbf{S}}_{\mathrm{I},2}$. The relative degrees of freedom associated with these impurity spins $\tilde{\mathbf{S}}_{\mathrm{I},1}$ and $\tilde{\mathbf{S}}_{\mathrm{I},2}$ are then integrated out, yielding a new "cluster" with total effective spin $S_{\mathrm{eff}} = |\tilde{\mathbf{S}}_{\mathrm{I},1} \mp \tilde{\mathbf{S}}_{\mathrm{I},2}|$, depending on the sign of J_{12}. This process modifies the effective interactions, yielding a new distribution $\rho_\Omega(J)$ of interactions between clusters that is dependent on the energy scale Ω. As Ω is successively lowered, the effective interactions between remaining spin clusters tend towards a fixed point power law distribution $\rho_\Omega(J) \sim J^{d/z-1}$, which gives rise to power law behaviour in relevant physical observables. Here, d is the effective dimension, and z is the dynamical critical exponent.

In order to derive approximate expressions for response in this scaling regime, it is useful to recast the summation over impurity spins as a summation over the clusters C.

$$\sum_m \langle \tilde{\mathbf{S}}_{\mathrm{I},m} \rangle = \sum_C^{N_C} \sum_{m \in C}^{n_C} \langle \tilde{\mathbf{S}}_{\mathrm{I},m} \rangle. \tag{46}$$

where $N_C(\Omega)$ denotes the total number of such clusters and n_C denotes the number of spins within the cluster C. As impurity spins become successively coupled at lower energies,

N_C decreases as Ω is lowered. At each energy scale Ω, it is assumed that the distribution $\rho_\Omega(J)$ is sufficiently broad, that each impurity cluster is approximated as an independent "spin", with effective moment size given by $S_{C,\text{eff}}$. As a result, each cluster is described by a thermodynamic partition function:

$$\mathcal{Z}_C = \frac{\sinh\left((2S_{C,\text{eff}}+1)\frac{\mu_\text{B}|\tilde{\mathbb{G}}_\text{I}\cdot\mathbf{H}|}{2k_\text{B}T}\right)}{\sinh\left(\frac{\mu_\text{B}|\tilde{\mathbb{G}}_\text{I}\cdot\mathbf{H}|}{2k_\text{B}T}\right)} \tag{47}$$

The contribution of each cluster to the torque is evaluated by taking the angular derivative of the free energy $G_C = -k_\text{B}T\ln\mathcal{Z}_C$, such that:

$$\tau = \sum_C^{N_C} \frac{\mathrm{d}G_C}{\mathrm{d}\theta} \tag{48}$$

This yields:

$$\frac{\tau}{H^2} = \frac{\mu_\text{B}}{H}\, g(\theta) \sum_C^{N_C} S_{C,\text{eff}}\, \mathcal{B}\left(S_{C,\text{eff}}, \frac{\mu_\text{B}|\tilde{\mathbb{G}}_\text{I}^\text{T}\cdot\mathbf{H}|}{k_\text{B}T}\right) \tag{49}$$

$$g(\theta) = -\left(\frac{\mathrm{d}\mathbf{h}^\text{T}}{\mathrm{d}\theta}\cdot\frac{\tilde{\mathbb{G}}_\text{I}\cdot\tilde{\mathbb{G}}_\text{I}^\text{T}}{|\tilde{\mathbb{G}}_\text{I}\cdot\mathbf{h}|}\cdot\mathbf{h}\right) \tag{50}$$

In order to simplify this expression further, we introduce the cluster average moment:

$$S_\text{eff}^\text{avg} = \frac{1}{N_C}\sum_C^{N_C} S_{C,\text{eff}}, \tag{51}$$

and approximate the cluster sum by:

$$\sum_C^{N_C} S_{C,\text{eff}}\mathcal{B}(S_{C,\text{eff}}, x) \approx N_C S_\text{eff}^\text{avg}\mathcal{B}(S_\text{eff}^\text{avg}, x). \tag{52}$$

Similarly, we identify the energy scale with the largest of either the thermal energy or typical Zeeman energy of a cluster:

$$\Omega = \max(k_\text{B}T, \mu_\text{B}S_\text{eff}^\text{avg}|\tilde{\mathbb{G}}_\text{I}^\text{T}\cdot\mathbf{H}|). \tag{53}$$

These expressions lead to the impurity torque expression given in the main text by Eq. (14). In practice, for the purpose of plotting, we use a soft maximum approximation, $\max(A, B) \approx (A^p + B^p)^{(1/p)}$ with $p = 2$. When plotted over several orders of A/B, the resulting functions are largely insensitive to the choice of p.

In order to evaluate the torque expression, the specific scaling of N_C and $S_{\text{eff}}^{\text{avg}}$ with Ω is required. This depends on the nature of the disordered fixed point[10,11]. At any given energy scale, the number of independent clusters scales as $N_C(\Omega) \sim \int_0^{\Omega} \rho_\Omega(J) dJ \sim \Omega^{d/z}$, which is an increasing function of Ω. For purely antiferromagnetic and unfrustrated interactions, the pairs of spins integrated out at any given energy scale would always form $S = 0$ singlets. As a result, no clusters of large moment would be formed as the energy is lowered, and $S_{\text{eff}}^{\text{avg}}$ would remain fixed. In contrast, in the presence of ferromagnetic or frustrated interactions[10,13], the average cluster moment must grow as Ω is successively lowered, scaling as $S_{\text{eff}}^{\text{avg}}(\Omega) \sim \Omega^{-\kappa}$ for some exponent κ. For purely ferromagnetic interactions, the average cluster moment would be directly proportional to the cluster size $S_{\text{eff}}^{\text{avg}} \propto N_C^{-1}$. For interactions with mixed signs, or frustrated antiferromagnetic couplings, the cluster moments increase more slowly $S_{\text{eff}}^{\text{avg}} \propto N_C^{-1/2}$, following the random walk argument of Supplementary Ref. 10 and 13. Therefore, for κ-Cu and other frustrated systems, this suggests $d/z = 2\kappa$ is applicable:

$$N_C(\Omega) = N_0 \Omega^{2\kappa} \tag{54}$$

$$S_{\text{eff}}^{\text{avg}}(\Omega) = S_0 \Omega^{-\kappa} \tag{55}$$

for some constants S_0 and N_0.

In the low temperature or high-field limit $k_{\text{B}}T \ll \mu_{\text{B}} S_{\text{eff}}^{\text{avg}} |\tilde{\mathbb{G}}_{\text{I}}^{\text{T}} \cdot \mathbf{H}|$, solving Supplementary Eq. (53) and (55) leads to:

$$\Omega \approx (\mu_{\text{B}} S_0 |\tilde{\mathbb{G}}_{\text{I}}^{\text{T}} \cdot \mathbf{H}|)^{\frac{1}{1+\kappa}}. \tag{56}$$

In this limit, all cluster moments should be saturated, leading to the relation for the magnetization $m \sim N_C S_{\text{eff}}^{\text{avg}} \propto H^{-\zeta_{\text{I}}+1} \propto H^{\kappa/(1+\kappa)}$. Thus, the relation between the exponents is given by:

$$\zeta_{\text{I}} = \frac{1}{1+\kappa} = \frac{2z}{2z+d} \leq 1. \tag{57}$$

Including prefactors, the reduced torque susceptibility follows from the definition of the torque as $\tau = \mu_{\text{B}}\, \mathrm{d}(\mathbf{H} \cdot \mathbf{m}_{\text{I}})/\mathrm{d}\theta$, together with the above expressions:

$$\tilde{\chi}_{\text{I}}^{H}(H) = \tilde{\chi}_{0,\text{I}}\ H^{-\zeta_{\text{I}}} \tag{58}$$

$$\tilde{\chi}_{0,\text{I}}^{H} = (2-\zeta_{\text{I}}) \frac{N_0 \mu_{\text{B}}^2 S_0^2}{(\mu_{\text{B}} S_0)^{\zeta_{\text{I}}}} \tag{59}$$

In the high temperature limit $k_{\mathrm{B}}T \gg \mu_{\mathrm{B}} S_{\mathrm{eff}}^{\mathrm{avg}} |\mathbb{G}_{\mathrm{I}}^{\mathrm{T}} \cdot \mathbf{H}|$ the energy scale is according to Supplementary Eq. (53):

$$\Omega \approx k_{\mathrm{B}}T. \tag{60}$$

With the scaling relations of N_C, Supplementary Eq. (54), and of $S_{\mathrm{eff}}^{\mathrm{avg}}$, Supplementary Eq. (55), and the relation between the exponent κ and ζ_{I}, Supplementary Eq. (57), the temperature dependence of the torque susceptibility, as given in Eq. (20) in the main text, follows from the expansion of the Brillouin function as $\mathcal{B}(S,x) = \frac{1}{3}(S+1)x$ for small x:

$$\tilde{\chi}_{\mathrm{I}}^{T}(T) \approx \tilde{\chi}_{0,\mathrm{I}}^{T} \left(\frac{1}{k_{\mathrm{B}}T} + S_0^{-1} (k_{\mathrm{B}}T)^{\frac{1}{\zeta_{\mathrm{I}}-2}} \right) \tag{61}$$

$$\tilde{\chi}_{0,\mathrm{I}}^{T} = \frac{2}{3} N_0 \mu_{\mathrm{B}}^2 S_0^2 \tag{62}$$

Of particular note, this expression (for suitable constants) reproduces the lowest two orders in the expressions for the susceptibiltiy derived in Supplementary Ref. 14 for random 1D chains.

Regarding the NMR linewidth, as discussed in Supplementary Ref. 15–17, the orphan spin impurities contribute through the staggered moment induced in the surrounding bulk around each impurity. As a result, a nuclear spin at site i in the bulk experiences a different effective local field, which is given by $\mathbf{H}_i = \mathbf{H} + \tilde{\mathbf{S}}_i \cdot \tilde{\mathbb{A}}_i$, in terms of the local hyperfine coupling tensor $\tilde{\mathbb{A}}$. We assume that the impurity-induced local magnetization is given by $\langle \tilde{\mathbf{S}}_i \rangle = a_i \langle \tilde{\mathbf{S}}_{\mathrm{I},m} \rangle$, where m labels the impurity closest to the dimer site i. The constants a_i are determined, for example, by the distance between i and m. Thus, finite impurity moments will lead to a distribution of local fields, which then broadens the NMR lines according to the specific distribution of a_i and $\langle \tilde{\mathbf{S}}_{\mathrm{I},m} \rangle$. An important observation is that the magnitude of this broadening depends explicitly only on local quantities, rather than the cluster averages appearing in the total impurity torque. This leads to a different scaling of the NMR linewidth ν with field and temperature. However, within a given cluster, the impurity moments are assumed to remain perfectly correlated. As a result, the external field is able to orient the local impurity spins only through coupling to the total moments of the clusters. These observations can be summarized by the approximation:

$$\nu \propto \frac{1}{\sqrt{N}} \left[\sum_{m}^{N} \langle \tilde{\mathbf{S}}_{\mathrm{I},m} \rangle \cdot \langle \tilde{\mathbf{S}}_{\mathrm{I},m} \rangle \right]^{\frac{1}{2}} \tag{63}$$

where the NMR linewidth scales as the root-mean-square impurity magnetization. Here, N is the total number of impurities. As before, we recast the summation in terms of clusters C:

$$\sum_{m}^{N}\langle\tilde{\mathbf{S}}_{\mathrm{I},m}\rangle\cdot\langle\tilde{\mathbf{S}}_{\mathrm{I},m}\rangle=\sum_{C}^{N_C}\sum_{m\in C}^{n_c}\langle\tilde{\mathbf{S}}_{\mathrm{I},m}\rangle\cdot\langle\tilde{\mathbf{S}}_{\mathrm{I},m}\rangle \tag{64}$$

where N_C gives the total number of clusters, and n_C gives the number of original impurity spins within cluster C. In analogy with Supplementary Eq. (49), the contribution per cluster is approximated by:

$$\sum_{m\in C}^{n_c}\langle\tilde{\mathbf{S}}_{\mathrm{I},m}\rangle\cdot\langle\tilde{\mathbf{S}}_{\mathrm{I},m}\rangle\approx n_C\left[\mathcal{B}\left(S_{C,\mathrm{eff}},\frac{\mu_{\mathrm{B}}|\tilde{\mathbb{G}}_{\mathrm{I}}^{\mathrm{T}}\cdot\mathbf{H}|}{k_{\mathrm{B}}T}\right)\right]^2 \tag{65}$$

Note that n_C appears as a prefactor here instead of $S_{C,\mathrm{eff}}$ due to the fact that $\langle\tilde{\mathbf{S}}_{\mathrm{I},m}\rangle\cdot\langle\tilde{\mathbf{S}}_{\mathrm{I},m}\rangle>0$. We then introduce that average cluster size as:

$$n_{\mathrm{avg}}=\frac{1}{N_C}\sum_{C}^{N_C}n_C \tag{66}$$

such that $n_{\mathrm{avg}}N_C=N$. Finally, making the approximation:

$$\sum_{C}^{N_C}n_C\left[\mathcal{B}\left(S_{C,\mathrm{eff}},x\right)\right]^2\approx n_{\mathrm{avg}}N_C\left[\mathcal{B}\left(S_{\mathrm{eff}}^{\mathrm{avg}},x\right)\right]^2 \tag{67}$$

provides to the proposed expression:

$$\nu_{\mathrm{I}}\approx\nu_0\ \mathcal{B}\left(S_{\mathrm{eff}}^{\mathrm{avg}},\frac{\mu_{\mathrm{B}}|\tilde{\mathbb{G}}_{\mathrm{I}}^{\mathrm{T}}\cdot\mathbf{H}|}{k_{\mathrm{B}}T}\right). \tag{68}$$

where $S_{\mathrm{eff}}^{\mathrm{avg}}$ appears only in the argument of the Brillouin function.

Finally, it is important to consider experimentally relevant values for the nonuniversal exponents appearing in the scaling forms. As noted above, the torque response of the orphan spin defects is parameterized by the nonuniversal exponent ζ_{I}, which is related to the low-energy distribution of effective interactions, $\rho_\Omega(J)\sim J^{2/\zeta_{\mathrm{I}}-3}$. Although ζ_{I} is unknown a priori, practical considerations restrict $1\lesssim z/d\leq\infty$, which corresponds to a narrow range $2/3\lesssim\zeta_{\mathrm{I}}\leq 1$. In general, ζ_{I} is likely to be sample dependent, and should tend to decrease with increasing frustration of the bulk interactions, and/or uniformity of the impurity distribution within the sample. Both such qualities lead to less singular fixed point distributions $\rho_\Omega(J)$. For example, the limit $\zeta\to 1$ (i.e. $z/d\to\infty$) corresponds to the infinite

randomness limit, in which $\rho_\Omega(J)$ is maximally singular. At any given energy scale, the vast majority of spins remain essentially decoupled, leading to a Curie-like response $\chi \sim 1/T$, up to logarithmic corrections. Such a fixed point describes, for example, the random singlet phase (RSP)[8,9] for purely antiferromagnetic but random interactions in $d = 1$. In this case, the interactions are not frustrated, and strongly interacting pairs of spins always form singlets, such that large spin clusters do not form at low energies, suggesting $\kappa = 0$. In contrast, the opposite limit of a flat energy distribution, given by $\zeta \to 2/3$ (i.e. $z/d \to 1$), corresponds to a so-called spin-glass fixed point (SGFP)[10]. This fixed point has been found in $d = 2$ via the SDRG appraoch for both geometrically frustrated lattices with random but purely antiferromagnetic interactions, as well as bipartite lattices with mixed ferro- and antiferromagnetic couplings[10]. In both cases clusters with large S_{eff} are generated at lower energies. Lying between these extremes are the so-called "large-spin" fixed points (LSFP), which have $0 < \kappa < 1/2$. For example, the inclusion of both ferro- and antiferromagnetic couplings in $d = 1$ leads to a LSFP[12–14] with $\kappa = 0.21$, $\zeta = 0.83$. Similarly, a LSFP also describes randomly site-diluted models in $d = 2$ with purely antiferromagnetic interactions, yielding $\kappa \sim 0.1-0.2$, depending on the degree of dilution. This corresponds to $\zeta \sim 0.8-0.9$.

In principle, the impurities in κ-Cu should correspond to a random $d = 2$ lattice with both site dilution and random ferro-/antiferromagnetic couplings. To the best of our knowledge, the appropriate exponents have not yet been studied for this case. However, it should be emphasized that a relatively large variation in z/d leads to narrow range of susceptibility exponents $2/3 \lesssim \zeta \leq 1$. On this basis, we conclude that the experimental values of $\zeta_{\mathrm{exp}} = 0.76 - 0.83$ observed for κ-Cu fall well within the range expected for impurity effects. The observed variance $\Delta\zeta = 0.07$ corresponds to about 20% of the realistic range, which may be an indication of strong sample dependence.

SUPPLEMENTARY REFERENCES

[1] Winter, S. M., Riedl, K. & Valentí, R. Importance of spin-orbit coupling in layered organic salts. *Phys. Rev. B* **95**, 060404 (2017).

[2] Neese, F. The orca program system. *Wiley Interdisciplinary Reviews: Computational Molecular Science* **2**, 73–78 (2012).

[3] Neese, F. Efficient and accurate approximations to the molecular spin-orbit coupling operator

and their use in molecular g-tensor calculations. *The Journal of chemical physics* **122**, 034107 (2005).

[4] Jeschke, H. O. *et al.* Temperature dependence of structural and electronic properties of the spin-liquid candidate κ-(BEDT-TTF)$_2$Cu$_2$(CN)$_3$. *Phys. Rev. B* **85**, 035125 (2012).

[5] Shekhtman, L., Entin-Wohlman, O. & Aharony, A. Moriya's anisotropic superexchange interaction, frustration, and Dzyaloshinsky's weak ferromagnetism. *Phys. Rev. Lett.* **69**, 836–839 (1992).

[6] Block, M. S., Sheng, D. N., Motrunich, O. I. & Fisher, M. P. A. Spin bose-metal and valence bond solid phases in a spin-1/2 model with ring exchanges on a four-leg triangular ladder. *Phys. Rev. Lett.* **106**, 157202 (2011).

[7] Holt, M., Powell, B. J. & Merino, J. Spin-liquid phase due to competing classical orders in the semiclassical theory of the Heisenberg model with ring exchange on an anisotropic triangular lattice. *Phys. Rev. B* **89**, 174415 (2014).

[8] Dasgupta, C. & Ma, S.-k. Low-temperature properties of the random Heisenberg antiferromagnetic chain. *Phys. Rev. B* **22**, 1305 (1980).

[9] Fisher, D. S. Random antiferromagnetic quantum spin chains. *Phys. Rev. B* **50**, 3799 (1994).

[10] Lin, Y.-C., Mélin, R., Rieger, H. & Iglói, F. Low-energy fixed points of random Heisenberg models. *Phys. Rev. B* **68**, 024424 (2003).

[11] Iglói, F. & Monthus, C. Strong disorder RG approach of random systems. *Physics reports* **412**, 277–431 (2005).

[12] Westerberg, E., Furusaki, A., Sigrist, M. & Lee, P. Random quantum spin chains: A real-space renormalization group study. *Phys. Rev. Lett.* **75**, 4302 (1995).

[13] Westerberg, E., Furusaki, A., Sigrist, M. & Lee, P. Low-energy fixed points of random quantum spin chains. *Phys. Rev. B* **55**, 12578 (1997).

[14] Frischmuth, B., Sigrist, M., Ammon, B. & Troyer, M. Thermodynamics of random ferromagnetic-antiferromagnetic spin-1/2 chains. *Phys. Rev. B* **60**, 3388 (1999).

[15] Shimizu, Y., Miyagawa, K., Kanoda, K., Maesato, M. & Saito, G. Emergence of inhomogeneous moments from spin liquid in the triangular-lattice Mott insulator κ-(ET)$_2$Cu$_2$(CN)$_3$. *Phys. Rev. B* **73**, 140407 (2006).

[16] Kolezhuk, A., Sachdev, S., Biswas, R. R. & Chen, P. Theory of quantum impurities in spin liquids. *Phys. Rev. B* **74**, 165114 (2006).

[17] Gregor, K. & Motrunich, O. I. Nonmagnetic impurities in a $S = 1/2$ frustrated triangular antiferromagnet: Broadening of ^{13}C NMR lines in κ-$(ET)_2Cu_2(CN)_3$. *Phys. Rev. B* **79**, 024421 (2009).
